# Use of dietary supplements by female seniors in a large Northern California health plan

**DOI:** 10.1186/1471-2318-5-4

**Published:** 2005-02-09

**Authors:** Nancy P Gordon, Donna M Schaffer

**Affiliations:** 1 Kaiser Permanente Medical Care Program, Oakland, California, USA

## Abstract

**Background:**

Women aged ≥ 65 years are high utilizers of prescription and over-the-counter medications, and many of these women are also taking dietary supplements. Dietary supplement use by older women is a concern because of possible side effects and drug-supplement interactions. The primary aim of this study was to provide a comprehensive picture of dietary supplement use among older women in a large health plan in Northern California, USA, to raise awareness among health care providers and pharmacists about the need for implementing structural and educational interventions to minimize adverse consequences of self-directed supplement use. A secondary aim was to raise awareness about how the focus on use of herbals and megavitamins that has occurred in most surveys of complementary and alternative therapy use results in a significant underestimate of the proportion of older women who are using all types of dietary supplements for the same purposes.

**Methods:**

We used data about use of different vitamin/mineral (VM) supplements and nonvitamin, nonmineral (NVNM) supplements, including herbals, from a 1999 general health survey mailed to a random sample of adult members of a large Northern California health plan to estimate prevalence of and characteristics associated with supplement use among women aged 65–84 (n = 3,109).

**Results:**

Based on weighted data, 84% had in the past 12 months used >1 dietary supplement, 82% a VM, 59% a supplement other than just multivitamin or calcium, 32% an NVNM, and 25% an herbal. Compared to white, nonHispanic women, African-Americans and Latinas were significantly less likely to use VM and NVNM supplements and Asian/Pacific Islanders were less likely to use NVNM supplements. Higher education was strongly associated with use of an NVNM supplement. Prevalence did not differ by number of prescription medications taken. Among white, nonHispanic women, multiple logistic regression models showed that college education, good health, belief that health practices have at least a moderate effect on health, and having arthritis or depression significantly increased likelihood of NVNM use, while having diabetes decreased likelihood.

**Conclusions:**

An extremely high proportion of older women are using dietary supplements other than multivitamins and calcium, many in combination with multiple prescription medications. Increased resources should be devoted to helping clinicians, pharmacists, supplement vendors, and consumers become more aware of the safety, effectiveness, and potential side effects of dietary supplements.

## Background

A recent national survey of medication use patterns found the highest prevalence of medication use over a one-week period was among women aged ≥ 65 years [1]. Over 80% of women in this age group had taken at least one prescription or over-the-counter medication during the week preceding the survey, and over half had taken five or more medications. In addition to these prescription medications, nearly 60% had used some type of vitamin/mineral supplement and 14% an herbal or other type of dietary supplement. These estimated prevalences of vitamin/mineral, herbal, and other supplement use are higher than those derived from previous national surveys [2-4], and by extrapolation, so is the prevalence of concomitant use of supplements and drugs. While many herbal medications and dietary supplements are safe for most people to use [5], there is growing evidence that some herbs and other types of non-herbal supplements can cause serious adverse effects [6-9]. Given the large proportion of this group concurrently using clinician-prescribed medications and self-prescribed dietary supplements, there is a great potential for drug-supplement interactions, especially since several surveys have shown that patients generally do not report or under-report use of supplements to their clinicians and pharmacists [10-13].

In a previous study, we used data from a 1999 general health survey of adult members of the Kaiser Permanente Medical Care Program of Northern California (KPCMP) to characterize use of nonvitamin, nonmineral (NVNM) supplements, including herbals, among the adult membership of this large health plan [14]. The results presented here expand on the earlier work by including vitamin/mineral (VM) supplement use, focusing on women aged 65–84, and employing logistic regression modeling to identify predictors of different types of dietary supplement use among these women. Our intent is to provide a comprehensive picture of dietary supplement use among this growing segment of the health care seeking population as a basis for planning structural and educational interventions to minimize adverse consequences of self-directed supplement use. A second aim is to raise awareness about how the focus on use of herbals and megavitamins that has occurred in most surveys of complementary and alternative therapy use results in a significant underestimate of the proportion of older women who are using all types of dietary supplements for the same purposes. This project was approved by the Kaiser Foundation Research Institute's Institutional Review Board in Oakland, CA.

## Methods

### Data sources

During Spring 1999, a confidential general health survey ("Adult Member Health Survey") was mailed to a stratified random sample of 40,000 adults aged ≥ 25 who were members of the Kaiser-Permanente Medical Care Program in Northern California. Up to three attempts were made to obtain a mailed response from each person in the sample unless the individuals refused or were deemed ineligible due to curtailment of membership, death, language barrier, or incorrect address. The survey questionnaire used in the first two mailing attempts included questions about use of complementary and alternative medicine (CAM) modalities and dietary supplement use, in addition to questions covering demographic and health-related characteristics and medication use. (A shortened form of the questionnaire sent to non-respondents to the first two mailings did not contain the CAM and dietary supplement questions.) Completed non-abridged questionnaires were received from 72% (n = 3,109) of women aged 65–84 in the survey sample. We restricted our estimates to women under age 85 because both the response rate and numbers of respondents aged ≥ 85 were too low to generalize about the "oldest old" cohort.

Use of dietary supplements during the previous 12 months was ascertained through two questions. First, a question covering use of 19 different CAM modalities: "Have you used the following methods to help treat or prevent health problems?" included items for "Any herbal medicine or supplement" and "Megavitamins/high dose vitamin therapy (not including daily multiple vitamin)." A separate question asked about use of selected dietary supplements: "In the past 12 months, did you use any nutritional supplements?" This question had a response checklist that included daily multiple vitamin with or without minerals (e.g., Centrum, One-a-Day), calcium with or without vitamin D, vitamin C, vitamin E, melatonin, *ginkgo biloba*, *Echinacea*, kava kava, glucosamine, St. John's Wort, and a space to write in other supplements, which were then individually coded and categorized as herbal supplements, other nonvitamin/nonmineral (NVNM) supplements, or vitamin/mineral (VM) supplements. Respondents were classified as VM users if they reported use of one or more vitamins/minerals, although we further subdivided this group into multivitamin and/or calcium users only and those who reported using other VMs such as vitamins C and E or minerals such as zinc and magnesium, with or without a daily multivitamin and/or calcium ("Dietary Supplement other than a Multivitamin and/or Calcium"). Respondents were classified as herbal users if they had indicated herbal use on the CAM modality checklist or indicated use of one or more herbals in the dietary supplement checklist. Respondents were classified as NVNM supplement users if they had indicated use of any of the herbals, glucosamine, or melatonin on the dietary supplement checklist or wrote in a supplement subsequently categorized as an herbal/botanical, amino acid, enzyme, protein, hormone or other non-herbal NVNM dietary supplement [15].

The estimated percentages of women who regular took one or more prescription medications were based on response to the question "How many prescription medicines do you regularly take," while estimated use of prescription medication with a narrow therapeutic index was based on health plan pharmacy data for the sample. The decision to use self-report rather than pharmacy data to estimate the numbers of prescription medications regularly was made because nearly 17% of the women had reported filling prescriptions outside of the health plan during the 12 months preceding the survey, and also because it was going to be extremely labor-intensive to determine from pharmacy data the numbers of different types of prescription medications each respondent "regularly" used.

### Statistical analysis

The respondent sample was assigned post-stratification weights so that analyses with weighted data would reflect the actual age (by 5-year intervals), gender and geographic distribution of the adult membership from which the sample was drawn. All percentages reported in the text and tables are based on weighted data. However, the tables include the actual (unweighted) subgroup denominators used in the analyses. All analyses were performed using PC-SAS version 8.2 [16]. Calculations of 95% confidence intervals (CI) and significance testing were done using the Proc Surveymeans procedure for data collected using a multi-stage survey design. The range of the confidence intervals is affected by the size of the subgroup denominator, which is why confidence intervals are tighter around prevalence estimates for the white, nonHispanic (whiteNH) subgroup than the estimates for the other race/ethnic subgroups.

Prevalence ratios (PR) were calculated to compare supplement use rates for subgroups of interest against rates for a reference group (e.g., herbal use among women aged 75–79 vs. aged 65–74). Confidence intervals around the PRs were used to assess the range of PR's compatible with the data at a level of 95% confidence. In the text and tables, a PR confidence interval that includes 1.0 indicates that rates are not statistically significantly different from each other at the p < .05 level. Logistic regression models run with unweighted data were used to test whether African-American/Black (AA/B), Hispanic/Latina (H/L), and Asian/Pacific Islander (A/PI) women differed significantly from white nonHispanic (whiteNH) women in use of different supplements after controlling for age and also after controlling for age, education, and health status.

Logistic regression models were used to identify statistically significant independent predictors of four types of use: (a) any dietary supplement, (b) any dietary supplement other than a multivitamin and/or calcium supplement only, (c) any NVNM supplement, and (d) any herbal supplement. The results we present are restricted to whiteNH women because we found that the predictor variables did not operate the same way in separately run models for the other race/ethnic groups in the sample. Indicator variables included in the logistic models were 3 age groups (75–79, 80–84 vs. 65–74), 4 education levels (< 12 years, some college, college graduate vs. high school graduate), overall health status (good/excellent health vs. fair/poor health), arthritis (yes vs. no), diabetes (yes vs. no), depression for at least two weeks during the past 12 months, and belief that "lifestyle/habits (what you eat, exercise, and weigh) affect health" moderately to extremely vs. not at all/a little bit. Rates of supplement use were not substantially different for ages 65–69 and 70–74, so these age groups were collapsed. All four logistic models included the same set of predictor variables and were run using data from the 96% (n=2378) whiteNH women who had complete data for all variables. In the text and tables, adjusted odds ratios (Adj. OR) with 95% confidence intervals that cross 1.0 are not statistically significant at the p < .05 level.

## Results and discussion

Table 1 shows selected demographic and health-related characteristics of the sample. The sample is predominantly whiteNH (80%), educated beyond high school (55%), and in good health (80%), although a large percentage have chronic health problems such as hypertension, diabetes, arthritis, and depression. Nearly 85% reported regularly taking at least one prescription medication and 19% five or more. Approximately 16% regularly used a prescription medication with a narrow therapeutic index, such as an anticoagulant, cardiac glycocide, or tricyclic antidepressant. Approximately 70% believed that their health habits had a moderate to large effect on their health.

**Table 1:** Characteristics of the sample population (n = 3109)

	N	Unwtd.%	Wtd.%*
			
All Ages			
Ages 65–74	1468	47.2	64.1
Ages 75–79	1356	43.6	22.9
Ages 80–84	285	9.2	13.0
			
Race/Ethnicity			
White, nonHispanic	2483	81.1	80.2
African-American/Black	169	5.5	5.7
Hispanic / Latina	147	4.8	5.0
Asian / Pacific-Islander	222	7.3	7.8
Other	41	1.3	1.2
			
Educational Attainment			
< High School Graduate	472	15.4	15.0
High School Graduate/GED	921	30.0	30.0
Some College	1090	35.6	34.8
4-Year College Graduate	583	19.0	20.2
			
Health Status			
Excellent/Very Good/Good	2436	78.7	79.6
Fair/Poor	658	21.3	20.4
			
Health Conditions			
Heart Disease	561	18.0	17.3
Diabetes	350	11.3	11.4
Hypertension	1449	46.6	46.4
Arthritis	1202	38.7	38.1
Depression for ≥ 2 weeks during yr	365	11.7	12.0
			
# Rx Medications Used (by self-report)			
1	500	16.9	16.8
2–4	1429	48.4	48.9
5 or more	567	19.2	19.1
Taking R_x _Medication with a Narrow Therapeutic Index**	513	16.5	15.9
			
Belief About How Much Health Habits/Lifestyle Affect Health			
Little or no effect	917	30.8	29.6
Moderate effect	591	19.8	19.1
Great deal of effect	1472	49.4	51.3

* Weighted percentages are based on respondent data weighted to reflect the age, gender, and geographic distribution of the membership. N's in table are actual numbers of respondents with this characteristic.

** Estimated based on health plan pharmacy records for sample.

Table 2 [See Additional file 1 ] shows estimates of the percentages of female seniors who used specific types of dietary supplements. Estimates are provided for all women and for women in the four major race/ethnic groups because the estimates for the overall population are so heavily influenced by the large proportion of whiteNH women in the sample. Overall, 84% of the women had used at least one dietary supplement during the previous 12 months, 82% a VM supplement, 59% a dietary supplement other than just a multivitamin and/or calcium, 32% an NVNM supplement, and 25% an herbal supplement. Among those who used at least one supplement (n = 2,574), a mean of 3.25 (sd = 15.29) supplements were used, with 22% using only one and 57.1% using ≥ 3 supplements. The mean number of supplements used excluding daily multivitamins and calcium was 2.14 (sd = 12.42), with 49% using only one and 27.3% using ≥ 3.

After adjusting for age, African-American/Black seniors were significantly less likely than whiteNH seniors to use daily multivitamins and calcium, as well as all the other categories of dietary supplements. This difference remained statistically significant after also adjusting for education, health status, and the three chronic health conditions. Hispanic/Latina seniors were also significantly less likely than whiteNH seniors to use VM supplements (with the exception of vitamin E) and NVNM supplements, but did not significantly differ on herbal use. Asian/Pacific Islander seniors did not significantly differ from whiteNH seniors on use of any type of dietary supplement, use of VMs, or use of dietary supplements other than just a multivitamin and/or calcium. However, they were significantly less likely than whiteNH women to use NVNM supplements. Among women using any supplement, mean use of all supplements and supplements excluding multivitamins and calcium were both significantly higher among whiteNH women than among women of color (not shown). Table 2 also shows that use of glucosamine by women with arthritis was significantly lower among African-American/Black, Hispanic/Latina, and Asian/PI women as compared to whiteNH women (OR_AA/B _= 0.32, CI: 0.13–0.82; OR_H/L _= 0.19, CI: 0.06–0.61; OR_A/P _= 0.56, CI: 0.28–1.11). Similarly, women of color who had experienced depression for at least two weeks during the 12-month interval were substantially less likely than whiteNHs to report using St. John's Wort to treat depression. Rates of *ginkgo biloba *use did not significantly differ by race/ethnicity.

Table 3 [See Additional file 2 ] shows how use of dietary supplements varied by personal characteristics other then race/ethnicity for the whole sample and Table 4 [See Additional file 3 ] provides this information for the whiteNH women who comprised most of the sample. The NVNM and herbal supplement use rates were lower for seniors aged 75–84 than for those aged 65–74, but the differences were smaller than those observed for race/ethnicity. While higher education was significantly associated with supplement use, it was more strongly associated with use of NVNM and herbal supplements than VM use. For example, NVNM use among college graduates was approximately 40% higher than that for high school graduates, while the rate for those who had not completed high school was approximately 40% lower than the rate for high school graduates. Health status and belief about the effect of health practices were more strongly associated with NVNM supplement use than use of any dietary supplement.

Table 5 [See Additional file 4 ] shows the results of multiple logistic regression models predicting use of any dietary supplement, use of any dietary supplement other than just a multivitamin or calcium, use of any NVNM supplement, and use of any herbal by whiteNH women. Women who had not completed high school were significantly less likely than high school graduates to use a dietary supplement other than daily multivitamin/calcium and an NVNM, but did not significantly differ in likelihood of using herbals. Higher education, especially a college degree, was associated with significantly higher likelihood of use of all four categories of supplements as compared to high school education. Good health remained significantly associated with use of dietary supplements other than multivitamin/calcium, NVNM use, and herbal use, although in the case with herbals, it was only significant after the three health conditions were entered into the model. Belief that health practices had a moderate-large effect on health was a significant predictor of both VM and NVNM use, even after controlling for its strong association with educational attainment. Having arthritis increased the likelihood of use of VMs and NVNMs, but not herbals. In contrast, having diabetes decreased the likelihood of using VM and NVNM supplements. Experiencing depression did not have a significant effect on use of VM supplements, but doubled the likelihood of use of NVNM supplements, especially herbals.

A logistic regression model predicting glucosamine use among women with arthritis found that being African-American/Black (OR = 0.42, CI: 0.16–1.08) or Hispanic/Latina (OR = 0.25, CI: 0.08–0.82) race/ethnicity significantly decreased the likelihood of glucosamine use as compared to whiteNH women, while having education beyond high school (OR = 1.64, CI:1.16–2.30) and the belief that health practices have a moderate-large effect on health (OR = 1.49, CI:1.03–2.15) were associated with significantly greater likelihood of glucosamine use.

The prevalence of use of NVNM supplements and dietary supplements other than a multivitamin and/or calcium did not differ by number of prescription medications taken (0, 1, 2–4, or ≥ 5) nor by whether a prescription medication with a narrow therapeutic index was being taken. Approximately 10% of women seniors who were taking an anticoagulant were also using *ginkgo biloba *or garlic supplements, even though there is evidence that this combination of blood thinners can lead to adverse consequences.

**Figure 1:**
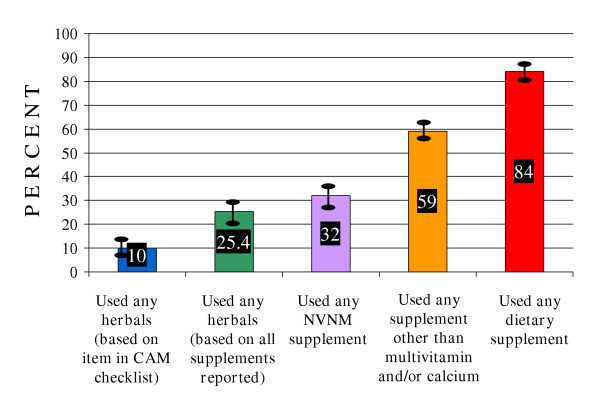
**Underestimation of dietary supplement use by tracking herbal use only among women aged 65–84**. NVNM = Nonvitamin, nonmineral including herbals. Based on respondent data weighted to reflect the age, gender, and geographic distribution of the membership.

Figure 1 shows how use of dietary supplements as a complementary or alternative therapy to treat or prevent health problems is probably underestimated among female seniors due to study investigators' focus on herbals and megavitamins. Prevalences of use of herbal and other dietary supplements were estimated based on responses provided by the same women to both the herbal supplement item in the complementary and alternative medicine (CAM) checklist and the nutritional supplement question. The estimated prevalence of herbal supplement based on reported specific supplements was twice as high as that based on the CAM checklist item alone. Broadening supplement use to include any NVNM significantly increased the percentage of users, primarily due to use of glucosamine for arthritis and other joint conditions. Further broadening to use of any NVNM or VM other than multivitamin and/or calcium resulted in a percentage twice as high as herbal use based on the coded supplements. Lastly, the prevalence of use of any type of VM or NVNM supplement, including more mainstream daily multiple vitamins and calcium, was more than three times greater than the prevalence of herbal use based on the coded supplements.

**Figure 2:**
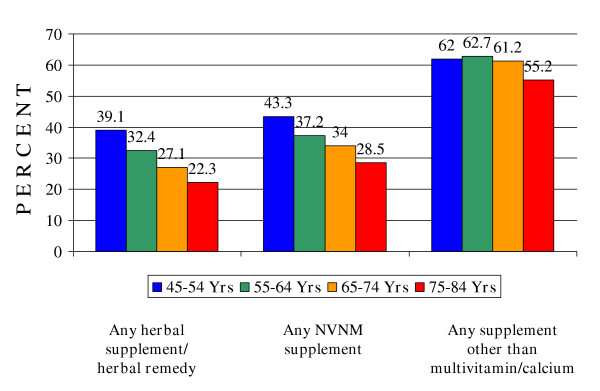
**Differences in dietary supplement use among women by age cohort in a health plan population**. NVNM = Nonvitamin, nonmineral supplement including herbals. Supplement other than Multivitamin/Calcium = any dietary supplement other than multivitamin and/or calcium. Based on respondent data weighted to reflect the age, gender, and geographic distribution of the membership.

Finally, Figure 2 shows estimated rates of NVNM supplement use among 45–54 and 55–64 year old women in this same health plan membership compared with rates of use among current seniors based on their responses to the same survey questions. Since current supplement use is probably one of the best predictors of future supplement use, the data suggest that the prevalence of NVNM use among women aged ≥ 65 years will increase substantially over the next couple of decades as the health plan population ages.

## Conclusions

The 84% prevalence of use of any vitamin/mineral (VM) or nonvitamin/nonmineral (NVNM) supplements, 32% prevalence of NVNM supplement use, and 25% prevalence of herbal supplement use by women aged 65–84 in this Northern California health plan membership are substantially higher than prevalences previously reported for women ≥ 65 years of age. Compared to the results of the national Slone Survey of medication use among adults, this population was significantly more likely to report use of any vitamin/mineral (82% vs. 59%), daily multivitamin (57% vs. 33%), calcium (57% vs, 23%), any herbal or other NVNM supplement (32% vs. 14%), and specific NVNM supplements, including *ginkgo biloba *(15% vs. 5%), glucosamine (12% vs. 4%), and *Echinacea *(8% vs. < 1%) [1]. While the time frame for the surveys differed (use during the past 12 months vs. use during the past week), this is unlikely to affect the comparison of most of the specific VM and NVNM supplements which are generally used almost daily. The percentages of herbal users (25%) and women using both herbals and prescription medicines (21%) were also higher than rates observed by Foster et al. (9% and 6%, respectively) based on Eisenberg *et al's *1997 national survey of alternative medicine use over a 12-month interval [17]. Further, our survey found that the 32% prevalence of NVNM use and 59% prevalence of use of NVNM or VM supplements other than just a multivitamin or calcium (59%) were substantially higher than prevalence of herbal use and were not affected by the number of prescription medications women were taking nor whether any of those medications had a narrow therapeutic index.

Our findings that supplement use was significantly higher among women who had higher levels of education, were white, nonHispanic (vs. African-American/Black or Hispanic/Latino), and were in good health are consistent with findings reported by other studies [18-22]. However, we also found that belief that one's health practices and lifestyle had at least a moderate effect on health was an additional significant predictor, but only for white, nonHispanic women; bivariate and logistic analyses done separately for African-American, Hispanic/Latino, and Asian/Pacific Islander subgroups found no indication that this factor influenced supplement use. For women of color, education beyond high school was the strongest predictor of use. Finally, we showed that some health conditions were significantly associated with higher likelihood of use of certain kinds of supplements (arthritis, depression), while another (diabetes) was associated with lower likelihood of use. This suggests that studies which employ one variable to represent presence of any chronic health problem may yield inaccurate results.

The higher rates we observed in our study may be a result of differences in the demographic composition of the survey populations. Our sample was predominantly (80%) white, nonHispanic and education beyond high school (35% some college and 20% college graduates), and in this and other surveys, being whiteNH and better educated was significantly associated with supplement use. However, while the usage rates among African-American/Black and Hispanic/Latina women and those without post-high school education are substantially lower, the demographics of the 1998–1999 Slone survey and 1997 Eisenberg *et al. *national survey samples are not substantially different from ours. Our observed usage rates may also be higher because of the social environment. Previous national surveys have shown that rates of NVNM and any alternative therapy use among people in the Western United States are higher than rates for the entire country [3,23,24]. However, the health plan membership is diverse with regard to education, socioeconomic status, and health-related attitudes.

The higher herbal and NVNM usage rates observed in this study compared to those reported by Foster *et al. *and Radimer *et al. *also may be related to the timing of the surveys. Eisenberg *et al. *reported highly significant increases in use of herbal medicine (2.5% to 12.1%) and megavitamin use (2.4% to 5.5%) for the adult population overall in1990 vs. 1997 [10]. In an earlier study, we reported an increase from 1.2% in 1996 to 9% in 1999 of use of herbals by women ≥ 65 years based on response to a question about use of different types of alternative therapies in this triennial health plan membership survey [25].

Finally, the wording of the questions to ascertain herbal use and NVNM use were not totally comparable across surveys. The estimates of VM and NVNM use reported in the Slone study are based on an open-ended question about use of any medication during the preceding 7 days, with a prompt for both VM and herbal supplement use, but not use of other types of NVNM supplements. Foster's estimate is based on response to a question about use of herbals as one of several different types of therapies. In contrast, our results are based on a question that provided a response checklist of some specific VM, herbal, and other NVNM supplements along with the opportunity for individuals to add additional supplements used, which were later coded and categorized. In an earlier study of alternative therapy use, we examined the difference in estimates of herbal use by the health plan membership based on indication of herbal use in a checklist of 17 different methods (not labeled as alternative therapies) used to treat or prevent health problems and indication of herbal use based on that item and response to the dietary supplement use question. We found that basing the estimate on the combined questions versus the single item nearly doubled the rate of herbal use among women ≥ 65 years (17.6% vs. 9.6%) [25].

Our finding that much larger percentages of adult women are taking NVNMs and VMs other than multivitamins and calcium suggests that for purposes of surveying populations about complementary and alternative therapy use and for medical interviews, the focus should be on use of all types of dietary supplements and medicinal teas, not just herbal supplements. There are several reasons for this. First, VMs and NVNMs other than herbals have the potential for causing adverse reactions, such as high doses of Vitamin C or zinc resulting in gastric upset, that can lead to further self-medication and/or medical visits. Second, certain VMs and NVNMs other than herbals also have the potential to interact with prescription medications, resulting in decreased effectiveness or affecting physiological indicators of how the medication is affecting the individual. Third, a focus on herbals alone excludes other NVNMs that are commonly used by patients with certain health conditions. For example, we estimate that nearly 3% of women used melatonin and 15% of whiteNH women (24% of those with arthritis) use glucosamine, neither of which is an herbal supplement. Finally, surveys of Mexican-American and Central American older women have shown a high prevalence of use herbal and other types of medicinal teas, which may not be picked up by questions asking about dietary supplement use [26]. However, since a growing proportion of the population is drinking herbal teas for nonmedicinal reasons (i.e., other than to treat health problems or symptoms), it will be important to find the best way to ask about medicinal tea use so as to avoid including in estimates those people who are drinking non-medicinal herbal teas as an alternative to caffeinated beverages.

Several surveys have found that patients do not tend to report use of herbals and other dietary supplements to their health care providers in clinical encounters [1,9,11,27]. Because of this lack of communication, there is a great potential for adverse interactions of drugs and dietary supplements in this age group. It may also be the case that some dietary supplements or particularly high dosages of supplements might actually cause symptoms or changes in physiological indicators that may be incorrectly attributed to other underlying health problems, resulting in unnecessary or inappropriate treatment either by the woman or her clinician. As the cost of prescription medications continues to rise and health insurers continue to place caps on medication coverage, it is likely that increasing numbers of older women, especially those on limited incomes, will turn to dietary supplements as a lower-cost alternative for treating health conditions. Concomitantly, the incidence of supplement-related health problems is likely to increase.

Our finding that nearly 60% of older women in this population were using dietary supplements other than multivitamins and calcium underscores the importance of clinicians querying patients about use of all types of dietary supplements when assessing health problems and prescribing medications, and as a back up, pharmacists inquiring about use of dietary supplements that may interact with prescription and over-the-counter medicines that are being purchased. Initiation of the communication by clinicians and pharmacists is likely to result in increased patient awareness that these dietary supplements may affect their health and treatment outcomes, which should then lead to higher rates of patient-initiated communication about dietary supplements they are using or considering using. Greater clinician and pharmacist awareness of all the different prescribed and self-directed regimens patients are using may lead to more proactive interventions to decrease adverse effects of supplement use. However, in order for clinicians and pharmacists to be able to respond to patient questions about dietary supplements, as well as to identify individuals at high risk for adverse effects, better information about the safety, effectiveness, and side-effects of dietary supplements need to be available and easily accessible, such as through the Natural Standard and National Medicines Databases.

In conclusion, our study indicates that use of dietary supplements to treat or prevent health problems is very prevalent among older insured women, and that based on current use in younger age groups, the prevalence can be expected to increase over the next few decades. It will be important for federal agencies, professional associations, manufacturers, and consumer groups to promote research into the safety and effectiveness of commonly used dietary supplements, to develop standards for product quality, and to develop guidelines for recommended dosages based on age, weight, and health history that can be disseminated to both health care professionals and stores or clinics which sell these products. At the same time, it is important to begin to educate patients and the broader public about the importance of more thoroughly researching the safety, effectiveness, and potential negative effects of particular dietary supplements before beginning to use them.

## Competing interests

The author(s) declare that they have no competing interests.

## Authors' contributions

NG designed the study, conducted the survey, analyzed the data, and drafted the manuscript. DS developed the scheme for coding the dietary supplement data, consulted on data analysis, and participated in manuscript development. Both authors read and approved the final manuscript.

## Pre-publication history

The pre-publication history for this paper can be accessed here:



## Supplementary Material

Additional File 1 Table 2 - Estimated percentages of female health plan members aged 65–84 using specific types of dietary supplements, overall and by race/ethnicity Click here for file

Additional File 2 Table 3 - Estimated use of dietary supplements by women aged 65–84 by selected personal characteristics other than race/ethnicity Click here for file

Additional File 3 Table 4 - Association of selected personal characteristics with dietary supplement use by white, nonHispanic women aged 65–84 Click here for file

Additional File 4 Table 5 - Results of multiple logistic regression models predicting dietary supplement use by white, nonHispanic women aged 65–84 Click here for file
