# Supplementary material for: Use of dietary supplements by female seniors in a large Northern California health plan
Source: BMC Geriatr. 2005 Feb 9;5:4. doi: 10.1186/1471-2318-5-4 (PMC549557; doi:10.1186/1471-2318-5-4)
Supplement: Additional File 1 — Table 2 - Estimated percentages of female health plan members aged 65–84 using specific types of dietary supplements, overall and by race/ethnicity [file 1471-2318-5-4-S1.doc]

**Table 2 - Estimated percentages of female health plan members aged 65-84 using specific types of dietary supplements,**

**overall and by race/ethnicity**

| Supplement Types | All Women  (n=3109) | White  (n=2483) | Afr.-Amer/Black  (n=169) | Latina  (n=147) | Asian/Pl  (n=222) |
| --- | --- | --- | --- | --- | --- |
|  | % (95% CI) | % (95% CI) | % (95% CI) | % (95% CI) | % (95% CI) |
| Any Vitamin/Mineral (VM) or Nonvitamin,  nonmineral (NVNM) dietary  supplement | 84.0 (± 1.4) | 85.8 (± 1.6) | 69.6 (± 7.8)^3^ | 73.3 (± 7.8)^3^ | 83.8 (± 5.3) |
| Any VM supplement | 82.2 (± 1.6) | 83.8 (± 1.6) | 68.8 (± 7.8)^3^ | 72.8 (± 7.8)^3^ | 82.8 (± 5.5) |
| Any dietary supplement other than a  daily multivitamin and/or calcium | 59.0 (± 2.0) | 61.8 (± 2.2) | 44.8 (± 8.4)^3^ | 44.0 (± 9.1)^3^ | 52.7 (± 7.5)^1^ |
| Any NVNM supplement | 32.0 (± 2.0) | 34.4 (± 2.2) | 18.1 (± 6.3)^3^ | 22.7 (± 7.6)^2^ | 22.7 (± 6.1)^2^ |
| Any herbal supplement | 25.4 (± 1.8) | 26.7 (± 2.0) | 16.4 (± 6.1)^2^ | 20.9 (± 3.8) | 18.9 (± 5.5) |
| Any protein, amino acid, enzyme,  hormone, or other nonherbal NVNM  dietary supplement | 16.6 (± 1.5) | 17.4 (± 1.8) | 4.7 (± 3.1)^3^ | 5.5 (± 3.9)^2^ | 7.2 (± 3.7)^2^ |
|  |  |  |  |  |  |
| Selected Specific Supplements |  |  |  |  |  |
| Daily multivitamin | 57.8 (± 2.0) | 59.5 (± 2.2) | 46.8 (± 8.4)^3^ | 48.0 (± 9.4)^2^ | 56.2 (± 7.2) |
| Calcium | 57.0 (± 2.0) | 59.3 (± 2.2) | 39.6 (± 8.4)^3^ | 46.9 (± 9.0)^2^ | 53.1 (± 7.5) |
| Vitamin C | 40.3 (± 2.0) | 41.8 (± 2.2) | 38.2 (± 8.2)^1^ | 27.4 (± 8.2)^2^ | 33.2 (± 7.0) |
| Vitamin E | 46.1 (± 2.0) | 48.2 (± 2.3) | 30.0 (± 8.0)^3^ | 40.3 (± 9.0) | 41.6 (± 7.0) |
| Vitamin B Complex | 2.7 (± 0.6) | 3.1 (± 0.8) | <1 -- | <1 -- | <1 -- |
| Zinc | 2.0 (+ 0.6) | 2.3 (± 0.8) | <1 -- | <1 -- | <1 -- |
| Echinacea | 8.3 (± 1.2) | 9.2 (± 1.4) | 3.6 (± 2.3)^1^ | 4.8 (± 3.9) | 5.1 (± 1.4) |
| Ginkgo biloba | 14.9 (± 1.4) | 15.5 (± 1.6) | 12.3 (± 5.7) | 11.9 (± 5.9) | 11.4 (± 4.3) |
| St. John's Wort (among women who  had been depressed 2+.weeks only) | 24.5 (± 5.3) | 27.3 (± 5.9) | 17.7 (±13.1) | 10.3 (± 9.4) | 4.9 (± 5.1)^1^ |
| Glucosamine | 12.7 (± 1.4) | 14.7 (± 0.8) | 4.2 (± 2.9)^3^ | 3.8 (± 3.5)^3^ | 5.6 (± 3.3) |
| (among women with arthritis only) | 21.6 (± 2.7) | 24.1 (± 3.1) | 9.0 (± 6.5)^1^ | 6.2 (± 7.4)^2^ | 13.2 (± 8.6) |
| Melatonin | 2.6 (± 0.6) | 2.8 (± 0.8) | 1.5 (± 2.2) | 1.4 (± 2.3) | 1.2 (± 1.6) |

Percentages are based on respondent data weighted to reflect the age, gender, and geographic distribution of the membership.

95% CI = 95% confidence intervals. If CI includes 1.0, the percentages are not statistically significantly different at p<.05.

^1^ p<.05; p<.01; ^3^ p<.001 indicates significance level for odds ratio comparing use of the supplement by women in the race/ethnic group

compared to white, nonHispanic women after adjusting for age in a logistic regression model.
