# Supplementary material for: Use of dietary supplements by female seniors in a large Northern California health plan
Source: BMC Geriatr. 2005 Feb 9;5:4. doi: 10.1186/1471-2318-5-4 (PMC549557; doi:10.1186/1471-2318-5-4)
Supplement: Additional File 2 — Table 3 - Estimated use of dietary supplements by women aged 65–84 by selected personal characteristics other than race/ethnicity [file 1471-2318-5-4-S2.doc]

**Table 3 - Estimated use of dietary supplements by women aged 65-84 by selected personal characteristics other than**

**race/ethnicity**

| Characteristic |  | Used Any VM or NVNM Supplement^†^ | Used any Dietary Supplement other than a Daily Multivitamin and/or Calcium^†^ | Used any NVNM Supplement^†^ | Used any Herbal Supplement |
| --- | --- | --- | --- | --- | --- |
|  | N | % (95% CI) | % (95%CI) | % (95%CI) | % (95%CI) |
| All Ages | 3109 | 84.0 (± 1.4) | 59.0 (± 2.0) | 32.0 (± 1.9) | 25.4 (± 1.8) |
| Ages 65-74 | 1468 | 84.8 (± 2.0) | 61.2 (± 2.5) | 34.0 (± 2.5) | 27.1 (± 2.3) |
| Ages 75-79 | 1356 | 82.0 (± 2.2) | 54.8 (± 2.8) | 28.7 (± 2.6) | 21.9 (± 2.3) |
| Ages 80-84 | 268 | 83.7 (± 4.5) | 55.8 (± 6.3) | 28.0 (± 6.0) | 23.1 (± 5.7) |
|  |  |  |  |  |  |
| Educational Attainment |  |  |  |  |  |
| < High School Graduate | 472 | 79.8 (± 3.9) | 43.0 (± 5.2) | 17.3 (± 3.9) | 14.0 (± 3.5) |
| High School Graduate | 921 | 80.6 (± 2.9) | 55.2 (± 3.7) | 28.5 (± 3.3) | 22.5 (± 3.1) |
| Some College | 1090 | 86.4 (± 2.3) | 64.1 (± 3.2) | 37.0 (± 3.3) | 28.9 (± 3.1) |
| 4-Year College Graduate | 583 | 89.0 (± 2.9) | 69.1 (± 4.2) | 40.0 (± 4.5) | 32.3 (± 4.3) |
|  |  |  |  |  |  |
| Health Status |  |  |  |  |  |
| Fair/Poor | 2436 | 80.7 (± 3.5) | 49.7 (± 4.5) | 24.0 (± 3.7) | 19.0 (± 3.3) |
| Good/Excellent | 658 | 84.8 (± 1.6) | 61.4 (± 2.2) | 33.9 (± 2.2) | 26.8 (± 2.0) |
|  |  |  |  |  |  |
| Health Conditions |  |  |  |  |  |
| Arthritis | 1202 | 87.1 (± 2.2) | 63.5 (± 3.1) | 37.0 (± 3.1) | 26.8 (± 2.9) |
| Diabetes | 350 | 74.4 (± 5.2) | 48.9 (± 6.1) | 24.8 (± 5.2) | 20.8 (± 5.0) |
| Hypertension | 1449 | 81.8 (± 2.3) | 56.3 (± 3.0) | 29.6 (± 2.7) | 22.4 (± 2.5) |
| History of Heart Problems | 561 | 82.8 (± 3.6) | 58.2 (± 4.8) | 29.8 (± 4.5) | 22.4 (± 4.1) |
| Depression for > 2 wks  during yr | 365 | 88.0 (± 3.5) | 67.5 (± 5.4) | 48.0 (± 5.8) | 44.3 (± 5.8) |
|  |  |  |  |  |  |
| Belief About How Much Health Habits/Lifestyle Affect Health |  |  |  |  |  |
| Not at all / A little | 917 | 79.3 (± 3.0) | 52.6 (± 3.8) | 25.0 (± 3.2) | 20.6 (± 3.0) |
| Moderately | 591 | 84.8 (± 3.2) | 58.6 (± 4.7) | 32.0 (± 4.4) | 26.1 (± 4.2) |
| A lot | 1472 | 87.7 (± 1.9) | 64.5 (± 2.8) | 37.3 (± 2.8) | 28.8 (± 2.7) |

^†^ VM=Vitamin and/or Mineral; NVNM = Nonvitamin, Nonmineral, including herbal, protein, amino acid, enzyme, and other unclassified

supplements such as glucosamine; Dietary supplements other than a daily multivitamin or calcium include Vitamin C, E, B complex,

zinc, etc., and all NVNM supplements.

Percentages are based on respondent data weighted to reflect the age, gender, and geographic distribution of the membership.

95% CI = 95% confidence intervals around the estimates
