# Supplementary material for: Use of dietary supplements by female seniors in a large Northern California health plan
Source: BMC Geriatr. 2005 Feb 9;5:4. doi: 10.1186/1471-2318-5-4 (PMC549557; doi:10.1186/1471-2318-5-4)
Supplement: Additional File 3 — Table 4 - Association of selected personal characteristics with dietary supplement use by white, nonHispanic women aged 65–84 [file 1471-2318-5-4-S3.doc]

**Table 4 - Association of selected personal characteristics with dietary supplement use by white, nonHispanic women aged 65-84**

| Characteristic |  | Used any VM or NVNM^†^  Supplement | | Used Any Dietary Supplement  other than a Daily Multivitamin and/or Calcium^†^ | | Used any NVNM Supplement^†^ | | Used any  Herbal Supplement | |
| --- | --- | --- | --- | --- | --- | --- | --- | --- | --- |
|  | N | % (95% CI) | PR^‡^ (95%CI) | % (95%CI) | PR^‡^ (95%CI) | % (95%CI) | PR^‡^ (95%CI) | % (95%CI) | PR^‡^ (95%CI) |
| Ages 65-74 (ref gp) | 1117 | 86.8 (± 2.1) | -- | 64.5 (± 2.9) | -- | 37.4 (± 2.9) | -- | 29.1 (± 2.8) | -- |
| Ages 75-79 | 1127 | 83.8 (± 2.2) | 0.96 (0.93-1.00) | 56.4 (± 3.1) | 0.87 (0.81-0.94)^3^ | 29.3 (± 2.9) | 0.78 (0.69-0.88)^3^ | 21.9 (± 2.6) | 0.75 (0.65-0.87)^3^ |
| Ages 80-84 | 239 | 84.8 (± 4.7) | 0.98 (0.92-1.04) | 59.2 (± 6.7) | 0.92 (0.81-1.04) | 30.3 (± 6.7) | 0.81 (0.64-1.02) | 24.5 (± 6.3) | 0.84 (0.64-1.11) |
|  |  |  |  |  |  |  |  |  |  |
| Educational Attainment |  |  |  |  |  |  |  |  |  |
| < High School Graduate | 309 | 83.1 (± 4.5) | 1.00 (0.94-1.07) | 46.9 (± 6.7) | 0.82 (0.70-0.96)^1^ | 19.3 (± 4.9) | 0.66 (0.49-0.87)^2^ | 15.6 (± 4.6) | 0.70 (0.50-0.97)^1^ |
| High School Graduate (ref gp) | 746 | 82.7 (± 3.1) | -- | 57.5 (± 4.0) | -- | 29.4 (± 3.8) |  | 22.4(± 3.5) | -- |
| Some College | 915 | 87.1 (±2.4) | 1.05 (1.00-1.10)^1^ | 65.1 (± 3.5) | 1.13 (1.03-1.24)^2^ | 38.4 (± 3.6) | 1.31 (1.12-1.53)^3^ | 29.2 (± 3.4) | 1.30 (1.07-1.58)^2^ |
| 4-Year College Graduate | 490 | 89.2 (±3.1) | 1.08 (1.02-1.14)^2^ | 71.4 (± 4.5) | 1.24 (1.13-1.37)^3^ | 43.8 (± 5.0) | 1.49 (1.25-1.77)^3^ | 35.2 (± 4.9) | 1.57 (1.27-1.94)^3^ |
|  |  |  |  |  |  |  |  |  |  |
| Health Status |  |  |  |  |  |  |  |  |  |
| Fair/Poor (ref gp) | 481 | 83.6 (± 3.7) | -- | 53.1 (± 5.3) | -- | 26.6 (± 4.6) |  | 20.5 (± 4.2) | -- |
| Good/Excellent | 1990 | 86.2 (±1.7) | 1.03 (0.98-1.08) | 63.7 (± 2.4) | 1.20 (1.08-1.33)^3^ | 36.0 (± 2.4) | 1.35 (1.13-1.62)^2^ | 27.9 (± 2.3) | 1.36 (1.08-1.72)^2^ |
|  |  |  |  |  |  |  |  |  |  |
| Belief About How Much Health Habits/Lifestyle Affect Health |  |  |  |  |  |  |  |  |  |
| Not at all/A little (ref gp) | 651 | 82.6 (±3.4) | -- | 55.3 (±4.5) | -- | 24.8 (± 3.8) |  | 19.8 (± 3.6) | -- |
| Moderately | 452 | 86.1 (± 5.0) | 1.04 (0.98-1.10) | 61.9 (± 5.3) | 1.12 (0.99-1.26) | 35.8 (± 5.3) | 1.44 (1.16-1.79)^3^ | 29.0 (± 5.1) | 1.46 (1.13-1.90)^2^ |
| A lot | 1286 | 88.0 (±2.0) | 1.06 (1.01-1.12)^1^ | 66.1 (±2.9) | 1.19 (1.09-1.31)^3^ | 39.5 (± 3.1) | 1.59 (1.33-1.90)^3^ | 30.0 (± 2.9) | 1.51 (1.24-1.86)^3^ |

^†^ VM=Vitamin and/or Mineral; NVNM = Nonvitamin, nonmineral, including herbal, protein, amino acid, enzyme, and other unclassified supplements such as

glucosamine; Dietary supplements other than a daily multivitamin or calcium include Vitamin C, E, B complex, zinc, etc., and all NVNM supplements

Percentages are based on respondent data weighted to reflect the age, gender, and geographic distribution of the membership.

95% CI = 95% confidence intervals around the estimates

PR = Prevalence ratios, a direct comparison of the rates (percentage/100) for one group to the rates for the reference group. Confidence intervals that

include 1.0 indicate that rates are not statistically significantly different at p<.05.

^1^ p<.05; ^2^ p<.01; ^3^ p<.001
