# Supplementary material for: Use of dietary supplements by female seniors in a large Northern California health plan
Source: BMC Geriatr. 2005 Feb 9;5:4. doi: 10.1186/1471-2318-5-4 (PMC549557; doi:10.1186/1471-2318-5-4)
Supplement: Additional File 4 — Table 5 - Results of multiple logistic regression models predicting dietary supplement use by white, nonHispanic women aged 65–84 [file 1471-2318-5-4-S4.doc]

**Table 5 - Results of multiple logistic regression models predicting dietary supplement use by white, nonHispanic women**

**aged 65-84**

| Characteristic | Used Any  VM or NVNM^†^  Supplement | Used Any Dietary Supplement other than Daily Multivitamin and/or Calcium | Used Any NVNM^†^  Supplement | Used Any Herbal  Supplement |
| --- | --- | --- | --- | --- |
|  | Adj. OR (95% CI) | Adj. OR (95% CI) | Adj. OR (95% CI) | Adj. OR (95% CI) |
| Ages 65-74 (ref gp) | -- | -- | -- | -- |
| Ages 75-79 | 0.79 (0.62-1.01) | 0.73 (0.61-0.88)^3^ | 0.72 (0.60- 0.87)^3^ | 0.75 (0.61-0.92)^2^ |
| Ages 80-84 | 0.81 (0.54-1.22) | 0.91 (0.67-1.23) | 0.81 (0.58-1.11) | 0.89 (0.63-1.26) |
|  |  |  |  |  |
| Educational Attainment |  |  |  |  |
| < High School Graduate | 0.90(0.63-1.29) | 0.64 (0.48-0.85)^2^ | 0.70 (0.50 – 0.98)^1^ | 0.74 (0.51-1.07) |
| High School Graduate/GED (ref gp) | -- | -- | -- | -- |
| Some College | 1.19 (0.90-1.57) | 1.23 (1.00-1.51) | 1.34 (1.08-1.67)^2^ | 1.25 (0.99-1.58) |
| College Graduate | 1.40 (0.97-2.00) | 1.51 (1.17-1.95)^2^ | 1.57 (1.22-2.03)^3^ | 1.60 (1.21-2.10)^3^ |
|  |  |  |  |  |
| Health Status  Good/Excellent vs. Fair/Poor (ref gp) | 1.23 (0.92-1.65) | 1.37 (1.09-1.71)^2^ | 1.38 (1.08-1.76)^1^ | 1.43 (1.09-1.88)^2^ |
|  |  |  |  |  |
| Arthritis (Yes vs. No) | 1.48 (1.16-1.88)^2^ | 1.53 (1.28-1.82)^3^ | 1.59 (1.33-1.91)^3^ | 1.10 (0.91-1.34) |
| Diabetes (Yes vs. No) | 0.48 (0.34-0.67)^3^ | 0.65 (0.49-0.86)^2^ | 0.72 (0.53-0.99)^1^ | 0.75 (0.53-1.06) |
| Depression for > 2 wks (Yes vs. No) | 1.15 (0.79-1.67) | 1.27 (0.97-1.66) | 2.09 (1.61-2.72)^3^ | 2.70 (2.06-3.52)^3^ |
|  |  |  |  |  |
| Belief About How Much Health Habits/ Lifestyle Affect Health |  |  |  |  |
| Not at all (ref gp) | -- | -- | -- | -- |
| Moderately | 1.19 (0.86-1.65) | 1.27 (0.98-1.63) | 1.47 (1.12-1.93)^2^ | 1.48 (1.10-1.99)^2^ |
| A lot | 1.40 (1.07-1.85)^1^ | 1.33 (1.08-1.63)^2^ | 1.53 (1.22-1.91)^3^ | 1.35 (1.05-1.72)^1^ |

^†^ VM=Vitamin and/or Mineral; NVNM = Nonvitamin, nonmineral, including herbal, protein, amino acid, enzyme, and other unclassified supplements

such as glucosamine; Dietary supplements other than a daily multivitamin or calcium include Vitamin C, E, B complex, zinc, etc., and all

NVNM supplements

* Odds ratios reflect increased or decreased likelihood of women with this characteristic using supplements compared to reference group after

adjusting for all other variables listed in the table using a multiple logistic regression model. Models were restricted to the 96% (n=2378)

of white nonHispanic women with no missing data.

^1^ p<.05; ^2^ p<.01; ^3^ p<.001
